# Two decades of surgical randomized controlled trials: worldwide trends in volume and methodological quality

**DOI:** 10.1093/bjs/znad160

**Published:** 2023-06-28

**Authors:** Aagje J M Pronk, Anne Roelofs, David R Flum, H Jaap Bonjer, Mohammed Abu Hilal, Marcel G W Dijkgraaf, Marc G Besselink, Usama Ahmed Ali

**Affiliations:** Department of Surgery, Amsterdam UMC, location University of Amsterdam, Amsterdam, the Netherlands; Cancer Centre Amsterdam, Amsterdam, the Netherlands; Department of Surgery, Amsterdam UMC, location University of Amsterdam, Amsterdam, the Netherlands; Cancer Centre Amsterdam, Amsterdam, the Netherlands; Department of Surgery, University of Washington, Seattle, Washington, USA; Cancer Centre Amsterdam, Amsterdam, the Netherlands; Department of Surgery, Amsterdam UMC, location Vrije Universiteit, Amsterdam, the Netherlands; Department of Surgery, Fondazione Poliambulanza Hospital, Brescia, Italy; Epidemiology and Data Science, Amsterdam UMC, location University of Amsterdam, Amsterdam, the Netherlands; Amsterdam Public Health, Amsterdam, the Netherlands; Department of Surgery, Amsterdam UMC, location University of Amsterdam, Amsterdam, the Netherlands; Cancer Centre Amsterdam, Amsterdam, the Netherlands; Department of Surgery, Amsterdam UMC, location University of Amsterdam, Amsterdam, the Netherlands; Cancer Centre Amsterdam, Amsterdam, the Netherlands

## Abstract

**Background:**

RCTs are essential in guiding clinical decision-making but are difficult to perform, especially in surgery. This review assessed the trend in volume and methodological quality of published surgical RCTs over two decades.

**Methods:**

PubMed was searched systematically for surgical RCTs published in 1999, 2009, and 2019. The primary outcomes were volume of trials and RCTs with a low risk of bias. Secondary outcomes were clinical, geographical, and funding characteristics.

**Results:**

Some 1188 surgical RCTs were identified, of which 300 were published in 1999, 450 in 2009, and 438 in 2019. The most common subspecialty in 2019 was gastrointestinal surgery (50.7 per cent). The volume of surgical RCTs increased mostly in Asia (61, 159, and 199 trials), especially in China (7, 40, and 81). In 2019, countries with the highest relative volume of published surgical RCTs were Finland and the Netherlands. Between 2009 and 2019, the proportion of RCTs with a low risk of bias increased from 14.7 to 22.1 per cent (*P* = 0.004). In 2019, the proportion of trials with a low risk of bias was highest in Europe (30.5 per cent), with the UK and the Netherlands as leaders in this respect.

**Conclusion:**

The volume of published surgical RCTs worldwide remained stable in the past decade but their methodological quality improved. Considerable geographical shifts were observed, with Asia and especially China leading in terms of volume. Individual European countries are leading in their relative volume and methodological quality of surgical RCTs.

## Teaser

In this systematic review, including 1188 surgical RCTs from 3 individual years in the past two decades (1999, 2009, and 2019), the worldwide volume remained stable in the past decade, whereas the rate of RCTs with a low risk of bias increased from 14.7 to 22.1 per cent, leaving ample room for improvement. The volume of published surgical RCTs increased in Asia, remained stable in North America, and decreased in Europe.

## Introduction

RCTs are essential in guiding clinical treatment decisions. However, conducting RCTs can be highly challenging given the numerous ethical, logistic, and financial hurdles. Furthermore, for RCTs to be worthwhile, they should meet high levels of methodological quality^1^. A weak or biased RCT may lead to abandonment of a beneficial intervention or the adoption of an ineffective intervention that might even harm patients^2^. Finally, an RCT must be reported in a clear and comprehensive manner to facilitate its interpretation and critical review.

Surgical RCTs have been criticized for their low methodological quality^3,4^. It is clear that such RCTs face some unique challenges, such as low patient accrual owing to strong patient and surgeon preferences, difficulties with blinding, steep surgical learning curves, varying surgical expertise and experience, variation in surgical quality control, and standardization of procedures^5^. A number of initiatives have been established to provide guidance in facing these unique challenges. Most notably, the IDEAL (Idea, Development, Exploration, Assessment and Long-term follow-up) collaboration formulated a framework to specifically evaluate complex interventions such as surgical procedures, including research options when an RCT might not be the appropriate study design^6,7^. To evaluate the status of surgical RCTs, the trends in volume and methodological quality of surgical RCTs published in 1999 and 2009 were assessed previously^8^. In recent years, however, new regulations such as the European Clinical Trials Directive have been put in place which may further hamper the execution of surgical RCTs^9–11^. The previous systematic review was updated with data from 2019 to assess trends in the volume and methodological quality of surgical RCTs in the past decade.

## Methods

**Fig. 1:**
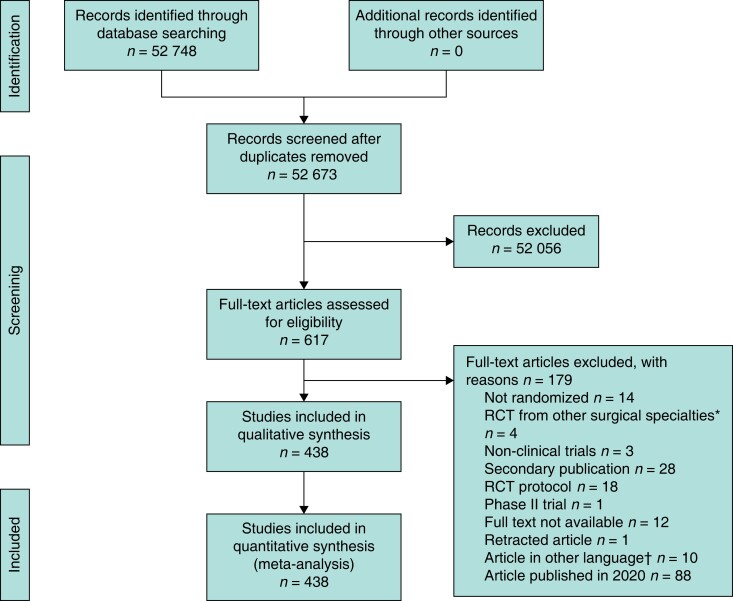
Flow chart showing selection of articles for review published in 2019 *?. †Six in Chinese, two in Russian, one in Portuguese, and one in Czech.

**Fig. 2:**
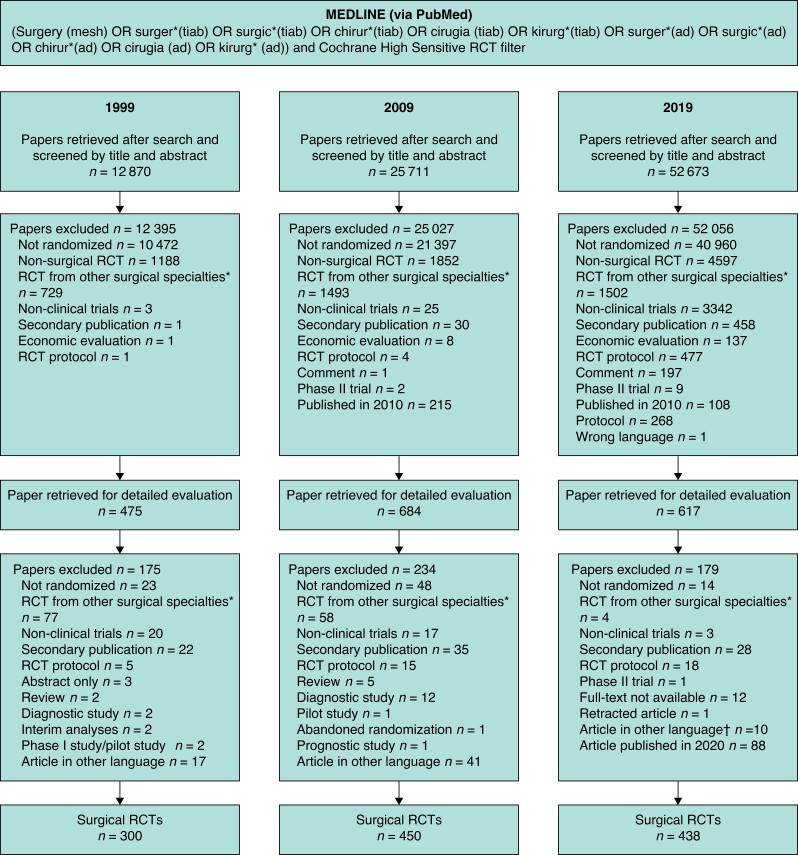
Flow chart of selection process for the years 1999, 2009, and 2019 *Including orthopaedics, urology, neurosurgery, cardiac surgery, ophthalmology, obtetrics and gynaecology, oral and maxillofacial surgery, and otolaryngology. †Six in Chinese, two in Russian, one in Portuguese, and one in Czech.

This review is a 10-year update of a previously published study^8^ and is reported in accordance with PRISMA guidelines (*Fig. 1*). Methodology was similar in regard to the search, inclusion and exclusion criteria, and data extraction^8^. In brief, the Cochrane High Sensitive Search Strategy was used augmented with free-text terms to identify RCTs in PubMed in 2019 (*Fig. 2*). Abstracts were screened for relevance by two reviewers and disagreements were solved by consensus between the two.

Inclusion and exclusion criteria were similar to those of the previous review^8^. A surgical RCT was identified as any trial determining the effect of a general surgical procedure (that is gastrointestinal, trauma based on affiliation, vascular, thoracic, breast, paediatric, transplantation, and other general surgical procedures, regardless of affiliation of corresponding author), or an RCT of which the corresponding author is affiliated to a general surgical department. If participants received an additional treatment (for example chemotherapy) as part of surgical treatment, the RCT was included. If the trial focused purely on the additional treatment and the corresponding author was not a surgeon, the RCT was excluded. RCTs published by other surgical specialties (cardiac surgery, neurosurgery, maxillofacial surgery, otolaryngology, ophthalmology, plastic surgery, gynaecology, urology and orthopaedics) were excluded^8^. Publications in languages other than English, French, German, and Dutch were excluded for practical reasons.

Clinical, geographical, and funding characteristics of included RCTs were extracted (*Table 1*). All RCTs were evaluated according to a nine-item list based on the Cochrane guidelines for methodological assessment of randomized trials: primary outcome; sample size calculation; presence of baseline; generation of allocation sequence; concealment of allocation; blinding; double blinding; type of analysis; and handling of drop-outs.

**Table 1:** Characteristics of included surgical randomized trials

	1999 (*n* = 300)	2009 (*n* = 450)	RR*†^	*P*†	2019 (*n* = 438)	RR*	*P~*
**Region**							
Africa/South America	6 (2.0)	31 (6.8)	3.44 (1.45, 8.16)	0.002	30 (6.8)	0.99 (0.61, 1.61)	1.000
Asia/Oceania	61 (20.3)	159 (35.3)	1.74 (1.34, 2.25)	<0.001	199 (45.4)	1.29 (1.09, 1.51)	0.002
Europe	161 (53.6)	204 (45.3)	0.84 (0.73, 0.98)	0.031	154 (34.2)	0.78 (0.66, 0.91)	0.002
North America	72 (24.0)	56 (12.4)	0.52 (0.38, 0.71)	<0.001	55 (12.6)	1.01 (0.71, 1.43)	1.000
**Countries**							
Single country (*versus* multinational)	273 (91.0)	408 (90.6)	1.04 (0.65, 1.64)	0.898	380 (86.8)	0.96 (0.91, 1.00)	0.071
**Centres**							
Single centre (*versus* multicentre)	173 (57.7)	270 (60.0)	1.04 (0.92, 1.18)	0.545	279 (63.7)	1.06 (0.96, 1.18)	0.269
**Specialty**							
Gastrointestinal surgery	156 (52.0)	203 (45.1)	0.87 (0.75, 1.01)	0.086	222 (50.7)	1.12 (0.98, 1.29)	0.107
Trauma	19 (6.3)	19 (4.2)	0.67 (0.36, 1.24)	0.234	16 (3.6)	0.87 (0.45, 1.66)	0.732
Vascular surgery	46 (15.3)	62 (13.8)	0.90 (0.63, 1.28)	0.596	34 (7.7)	0.56 (0.38, 0.84)	0.004
Other‡	79 (26.3)	166 (36.9)	1.40 (1.12, 1.75)	0.003	166 (37.9)	1.03 (0.87, 1.22)	0.782
**Malignancy**							
Benign disease§	196 (65.3)	287 (63.8)	0.98 (0.88, 1.09)	.697	223 (50.9)	0.80 (0.71, 0.90)	<0.001
Malignant disease§	80 (26.7)	102 (22.7)	0.85 (0.66, 1.10)	.224	160 (36.5)	1.61 (1.31, 1.99)	<0.001
Both	6 (2.0)	18 (4.1)	2,00 (0,80, 4,98)	0.143	48 (11.0)	2.74 (1.62, 4.63)	<0.001
Unclear	18 (6.0)	43 (9.6)	1.59 (0.94, 2.71)	0.101	7 (1.6)	0.17 (0.08, 0.37)	<0.001
**Type of intervention studied**							
Surgical procedure	112 (37.3)	212 (47.1)	1.26 (1.06, 1.51)	0.009	243 (55.5)	1.18 (1.04, 1.34)	0.013
Medication	133 (44.3)	144 (32.0)	0.72 (0.60, 0.87)	0.001	66 (15.1)	0.47 (0.36, 0.61)	<0.001
Other	55 (18.3)	94 (20.9)	1.14 (0.85, 1.54)	.402	129 (29.5)	1.41 (1.12, 1.78)	0.003
**Type of reference intervention**¶							
Similar surgery	71 (63.3)	138 (65.1)	–	0.752	173 (71.2)	–	0.005
Different surgery	31 (27.7)	50 (23.5)			62 (25.5)		
Non-surgical invasive	2 (1.8)	7 (3.3)			3 (1.2)		
Non-surgical non-invasive	8 (7.1)	17 (8.0)			5 (2.1)		
**Specialty of journal**							
Surgical journals	177 (59.0)	240 (53.3)	0.90 (0.80, 1.03)	.134	214 (48.9)	0.92 (0.81, 1.04)	0.202
**Impact factor#**	274 (91.3)	375 (83.3)			426 (97)		
Median (i.q.r.)	2.24 (1.13– 3.00)	2.57 (1.86– 3.72)	–	<0.001	2.80 (1.68– 4.51)	–	0.048
**Journal rank#**							
Top 10 surgery	29 (9.7)	56 (12.4)	1.41 (0.92, 2.14)	0.126	53 (12.1)	0.81 (0.57, 1.15)	0.919
Top 10 general	14 (4.7)	8 (1.7)	0.42 (0.18, 0.98)	0.048	10 (2.3)	1.07 (0.43, 2.69)	0.640
**Trial design**							
Parallel (*versus* crossover)	300 (100)	435 (96.7)	1.01 (0.98, 1.04)	0.503	431 (98.4)	1.02 (1.00, 1.04)	0.477
**Sample size, median (i.q.r.)**	70 (40–151)	78 (47–141)	–	0.233	10 050 (64– 190)	–	<0.001
**Funding**							
Industry funded	68 (22.7)	83 (18.4)	0.81 (0.61, 1.08)	0.164	50 (11.4)	0.62 (0.45, 0.86)	0.003
Non-industry funded	61 (20.3)	177 (39.3)	1.93 (1.50, 2.49)	<0.001	236 (53.9)	1.37 (1.19, 1.58)	<0.001
Not reported	171 (57.0)	190 (42.2)	0.74 (0.64, 0.86)	<0.001	152 (34.7)	0.82 (0.70, 0.97)	0.021
**Funding reported+**							
Industry funded	68 (52.7)	83 (31.9)	0.61 (0.48, 0.77)	<0.001	50 (17.5)	0.55 (0.40, 0.75)	<0.001
Non-industry funded	61 (47.2)	177 (68.0)	1.44 (1.18, 1.76)	<0.001	236 (82.5)	1.21 (1.10, 1.34)	<0.001

Values are *n* (%) unless indicated otherwise; *values in parentheses are 95% confidence intervals. †This comparison was performed in the authors’ previous study8. +Analysis excluding the less informative ‘not reported’ group was added to aid interpretation of results. The relationship observed over time regarding industry funding persisted and relative differences were more pronounced. ‡Includes breast, abdominal wall, thoracic, and endocrine surgery. §Analysis excluding the less informative ‘unclear’ group showed similar results regarding the trend for benign and malignant disease over time. ¶For trials studying surgical interventions only. #On the basis of impact factors of the Institute for Scientific Information for the respective year (1999, 2009, 2019). Journals without an impact factor are not included. ^Comparing 1999 and 2009 using Fisher’s exact, χ^2^, and Mann–Whitney U tests. ~Comparing 2009 and 2019 using Fisher’s exact, χ^2^, and Mann–Whitney U tests. RR, relative rate.

Detailed definitions used for each item have been published previously^8^. A trial with a low risk of bias was defined as one that met all of the following four requirements: adequate generation of allocation, adequate concealment of allocation, intention-to-treat analysis, and adequate handling of drop-outs. Extraction of all data was conducted by two reviewers, and all discrepancies were reviewed by one of the senior authors.

Characteristics of the trials were compared between each pair of consecutive study years (1999 *versus* 2009 and 2009 *versus* 2019). A subgroup analysis was performed for volume and quality based on the geographical area of origin. For studies published in 1999, 2009, and 2019, population data from the years 2000, 2010, and 2019 respectively were used^12–14^. Median (i.q.r.) values were calculated for continuous data, whereas dichotomous outcomes are presented as the number of events with percentage. Data from 2009 were compared with data from 1999, and data from 2019 with those from 2009, by Fisher’s exact, χ^2^, and Mann–Whitney *U* tests, and the relative rate (RR) with corresponding 95 per cent confidence interval, as appropriate. *P* < 0.050 was considered statistically significant.

## Results

The search for 2019 was undertaken on 3 April 2020 and identified 52 673 PubMed hits (*Fig. 1*). The search in 1999 and 2009 was performed on 3 June 2010 and identified 12 870 and 25 611 PubMed hits respectively. After screening, 300, 450, and 438 surgical RCTs published in 1999, 2009, and 2019 respectively were identified (*Fig. 2*).

### General characteristics

Epidemiological and clinical characteristics of the included trials are shown in *Table 1*. The median sample size increased during the study, from 78 patients in 2009 to 100 patients per trial in 2019 (*P* < 0.001). Reports of multicentre trials (42.3, 40.0, and 36.3 per cent in 1999, 2009, and 2019 respectively; *P* = 0.257) and international trials (9.0, 9.4, and 13.2 per cent; *P* = 0.065*)* did not change significantly over time. In 2019, gastrointestinal/oncological surgery was the most common specialty, accounting for 50.7 per cent of all surgical RCTs, compared with 7.7 per cent for vascular surgery, and 3.6 per cent for trauma surgery. Of the published RCTs, 36.5 per cent addressed malignant diseases. There was a small increase in trials studying a surgical procedure, from 212 in 2009 to 243 in 2019 (RR 1.18, 95 per cent c.i. 1.04 to 1.34; *P* = 0.013). Most of these trials compared two different surgical procedures. The proportion of industry-funded trials almost halved from 18.4 per cent in 2009 to 11.4 per cent in 2019 (RR 0.62, 0.45 to 0.86; *P* = 0.003). Concomitantly, there was a significant absolute increase of 33.3 per cent (RR 1.37, 1.19 to 1.58; *P* < 0.001) in investigator-initiated (non-industry) trials. In an analysis excluding trials lacking informative data (that is, not reported), the decrease in industry-funded and increase in non-industry trials was clearer and more pronounced (*Table 1*). The number of trials not reporting the source of funding decreased every year, being 57.0 per cent in 1999, 42.2 per cent in 2009, and 34.7 per cent in 2019.

### Volume

Overall, the absolute volume of RCTs remained stable between 2019 and 2009 (438 *versus* 450). This contrasts with a 50.0 per cent increase in the previous decade (300 RCTs in 1999). In 2019, most RCTs originated from Asia/Oceania (*Table 1*). There was an increase in absolute and relative volume of RCTs from this region compared with 2009. However, the increase in volume in 2009–2019 (25.2 per cent) was smaller than in 1999–2009 (160.7 per cent). In contrast, Europe showed a decrease in volume of RCTs between 2019 and 2009 (154 and 204 RCTs). The volumes remained virtually the same in North America (55 and 56 RCTs) and Africa/South America (30 and 31 RCTs) for 2019 and 2009 respectively.

In 2019, China was the country with the largest volume of surgical RCTs (*Table 2*). There was an increase in both absolute and relative volume, from 40 trials (8.9 per cent) in 2009 to 81 (18.5 per cent) in 2019. The volume of published trials in the USA remained stable in the past decade, with 50 in 2019 (*Table 2*). When the number of inhabitants was considered, Finland was the country with the most trials relative to population in 2009 and 2019, with the Netherlands in second place in both years.

**Table 2:** Top 10 countries by absolute and relative volume of published surgical randomized trials

Top 10 by absolute volume of surgical RCTs*				Top 10 by relative volume of surgical RCTs per 10 million inhabitants			
Rank	1999 (*n* = 300)	2009 (*n* = 450)	2019 (*n* = 438)	Rank	1999 (*n* = 300)	2009 (*n* = 450)	2019 (*n* = 438)
1	USA 65 (21.7)	USA 52 (11.6)	China 81 (18.5)	1	Denmark 23.9	Finland 19.0	Finland 23.5
2	Italy 31 (10.3)	China 40 (8.9)	USA 50 (11.4)	2	Finland 17.1	Netherlands 10.8	Netherlands 15.8
3	UK 30 (10.0)	UK 39 (8.7)	Japan 36 (8.2)	3	Sweden 15.5	Ireland 10.6	Denmark 10.4
4	Japan 24 (8.0)	Italy 37 (8.2)	Netherlands 27 (6.2)	4	Netherlands 8.0	Sweden 9.9	Switzerland 9.3
5	Germany 21 (7.0)	Germany 34 (7.6)	Korea 20 (4.6)	5	Australia 5.8	Switzerland 9.1	Norway 9.3
6	Sweden 14 (4.7)	Japan 26 (5.8)	Egypt 18 (4.1)	6	Switzerland 5.5	Austria 7.3	Sweden 8.0
7	Denmark 13 (4.3)	Turkey 26 (5.8)	Italy 16 (3.7)	7	Italy 5.4	Greece 6.5	Estonia 7.5
8	Netherlands 13 (4.3)	India 19 (4.2)	UK 15 (3.4)	8	UK 5.1	UK 6.2	Lithuania 7.2
9	Australia 11 (3.7)	Netherlands 18 (4.0)	Spain 15 (3.4)	9	Singapore 4.6	Italy 6.0	Bahrain 6.09
10	Finland 9 (3.0)	Egypt 15 (3.3)	Finland 13 (3.0)	10	Greece 4.5	Norway 4.2	Bosnia 6.05

*Values are *n* (%).

### Reported risk of bias

Methodological characteristics related to the risk of bias of the included trials are shown in *Table 3*. In 2019, more trials described a sample size calculation, adequate methods for generation and concealment of allocation, and the use of any type of blinding than in 2009 (*P* ≤ 0.001 for all). In contrast, fewer trials reported adequate handling of drop-outs in 2019. Reporting of primary outcome and baseline characteristics did not change significantly over time. Blinding remained problematic, with only 41.3 per cent of trials having any type of blinding and only 16.2 per cent being double blind. The proportion of RCTs with a low risk of bias increased from 14.7 per cent in 2009 to 22.1 per cent in 2019 (RR 1.51, 95 per cent c.i. 1.14 to 2.01; *P* = 0.004). Methodological quality characteristics by geographical region are shown in *Table S1*.

**Table 3:** Reported methodological quality of included surgical randomized trials

	1999 (*n* = 300)	2009 (*n* = 450)	RR*†	*P*†^	2019 (*n* = 438)	RR*	*P~*
Primary outcome stated explicitly	203 (67.7)	297 (66.0)	0.98 (0.88, 1.08)	0.693	315 (71.9)	0.92 (0.84, 1.00)	0.057
Sample size calculation described	101 (33.7)	218 (48.4)	1.44 (1.20, 1.73)	<0.001	296 (61.4)	0.72 (0.64, 0.80)	<0.001
Baseline present	272 (90.7)	414 (92.0)	1.02 (0.97, 1.06)	0.594	412 (94.1)	0.98 (0.94, 1.01)	0.228
**Generation of allocation: reported and adequate**	96 (32.0)	213 (47.3)	1.48 (1.22, 1.79)	<0.001	283 (64.6)	1.49 (1.28, 1.74)	<0.001
Computer	52 (17.3)	138 (30.7)			212 (48.4)		
Random table	24 (8.0)	30 (6.7)			41 (9.4)		
Other adequate	20 (6.7)	45 (10.0)			30 (6.8)		
**Concealment of allocation: reported and adequate**	96 (32.0)	224 (50.0)	1.56 (1.29, 1.88)	<0.001	287 (65.5)	0.76 (0.68, 0.85)	<0.001
Central/pharmacy	32 (10.7)	60 (13.3)			88 (20.1)		
Envelopes	57 (19.0)	145 (32.2)			140 (32.0)		
Other adequate	7 (2.3)	19 (4.2)			59 (13.5)		
Blinding: any type of blinding	103 (34.3)	138 (30.7)	0.89 (0.73, 1.10)	0.300	181 (41.3)	0.74 (0.62, 0.89)	0.001
Double blinding stated	72 (24.0)	90 (20.0)	0.83 (0.63, 1.10)	0.205	71 (16.2)	0.81 (0.61, 1.07)	0.143
**Type of analyses**							
Intention to treat	60 (20.0)	149 (33.1)	1.66 (1.27, 2.15)	<0.001	156 (35.6)	1.08 (0.90, 1.29)	0.432
Per protocol	9 (3.0)	16 (3.6)			14 (3.2)		
Not stated	231 (77.0)	285 (63.3)			268 (61.2)		
**Handling of drop-outs adequate**	253 (84.3)	373 (82.9)	1.01 (0.95, 1.07)	0.836	282 (64.4)	0.78 (0.72, 0.84)	<0.001
Low risk of bias	17 (5.7)	66 (14.7)	2.59 (1.55, 4.32)	<0.001	97 (22.1)	1.51 (1.14, 2.01)	0.004

Values are *n* (%) unless indicated otherwise; *values in parentheses are 95% confidence intervals. †This comparison was performed in the authors’ previous study^8^. ^Comparing 1999 and 2009 using Fisher’s exact, χ^2^, and Mann–Whitney U tests. ~Comparing 2009 and 2019 using Fisher’s exact, χ^2^, and Mann–Whitney U tests. RR, relative rate.

In the interval 2009–2019, there was a significant increase in trials with a low risk of bias in Asia/Oceania (from 4.9 per cent in 2009 to 18.1 per cent in 2019; RR 3.50, 1.70 to 7.32; *P* < 0.001). In contrast, RCTs from Africa/South America did not show improvement in reported methodological characteristics over the past 10 years; the proportion of trials with a low risk of bias was below 10 per cent in both years. Quality did not significantly improve in Europe (from 23.0 per cent in 2009 to 30.5 per cent in 2019; RR 1.35, 0.96 to 1.92; *P* > 0.050) and North America (from 16.1 per cent in 2009 to 23.6 per cent in 2019; RR 1.47, 0.69 to 3.16; *P* > 0.050) (*Table S1*). The top 10 countries by methodological quality in 2019 are shown in *Table 4*. The top three countries were all in Europe; The UK had the highest proportion of trials with a low risk bias of (57.1 per cent), with the Netherlands ranking second (51.9 per cent), and Finland ranking third (38.5 per cent). Korea was in fourth place (30.0 per cent). Nigeria was the top country by relative number of RCTs per specialist surgical workforce per 100 000 inhabitants (*Table S2*).

**Table 4:** Top 10 ranking for countries in 2019 based on the proportion of trials with a low risk of bias

	Trials with low risk of bias (%)†	No. of trials	Rank on the basis of no. trials per 10^7^ inhabitants	Impact factor*	Adequate generation of allocation	Adequate concealed allocation	Intention-to-treat analysis	Adequate handling of drop-outs
1 UK	57.1	15	19	3.37 (2.02–21.94)	93.3	73.3	66.7	73.3
2 Netherlands	51.9	27	2	4.28 (2.72–14.78)	85.2	88.9	66.7	77.8
3 Finland	38.5	13	1	4.50 (2.48–10.48)	61.5	92.3	53.9	76.9
4 Korea	30.0	20	44	2.11 (1.47–4.12)	85.7	60.0	43.8	80.0
5 Germany	27.3	11	27	4.84 (3.18–6.26)	63.6	54.6	72.7	81.8
6 Spain	26.7	15	15	3.15 (2.20–5.68)	86.7	73.3	46.7	73.3
7 USA	22.0	50	24	3.61 (1.98–8.76)	60.0	70.0	36.0	70.0
8 Japan	20.0	35	16	2.39 (1.88–6.08)	54.3	51.4	34.3	77.1
9 China	16.0	81	39	2.24 (1.70–3.65)	59.3	51.9	21.1	55.6
10 Italy	12.5	16	17	2.00 (0.77–5.53)	62.5	56.3	25.0	68.8

*Values are median (i.q.r.) 2019 impact factors. Only countries with at least 10 published trials were analysed. †Trial with adequate generation of allocation, adequate concealment of allocation, intention-to-treat analyses, and adequate handling of drop-outs.

## Discussion

This updated systematic review demonstrated that the overall worldwide volume of published surgical RCTs has remained stable in the past decade. This in contrast to a 50.0 per cent increase a decade earlier. The region of origin of published surgical RCTs has shifted considerably, with Asia/Oceania now the leading continent in volume, with 45.4 per cent of surgical RCTs, whereas the number of RCTs from Europe has declined. China has become the leading country in terms of absolute volume, followed by the USA. Concerns about methodological quality persist, although almost all quality characteristics have improved. Some 94.3 per cent of RCTs were at moderate or high risk of bias two decades ago, whereas this has now decreased to 77.9 per cent. Certain European countries continue to be leading in terms of relative RCT volume per capita (Finland, the Netherlands) and methodological quality (UK, the Netherlands).

Although the volume of published surgical RCTs has decreased slightly in the past decade, the overall volume of published (both surgical and non-surgical) RCTs continued to increase between 1966 and 2018 (176 620 trials)^15^. This can be explained partly by the described difficulties associated with performing surgical RCTs. For example, when two interventions have different benefit-to-harm profiles, patients and surgeons may have strong treatment preferences^16^. This may lead to difficulties in recruitment of both patients as well as surgeons, especially for a complex surgical procedure in which surgical experience differs^17^. This might hinder recruitment and also pose a threat to external RCT validity. These difficulties might drive researchers to opt for other study designs, such as prospective cohort studies, resulting in fewer RCTs than in other specialties^18,19^. To overcome treatment preferences, participating surgeons should have clearly passed the learning curve and be able to perform both techniques. There may also be different surgeons from one unit^16,20^.

However, the slight decrease in volume of surgical RCTs over the past decade is not necessarily a negative development. An ever-increasing number of RCTs cannot be the ultimate goal. The results of this review may indicate that the steady state has been reached. An improvement in this respect is that trial quality has improved significantly over the past decade, while the size has remained the same. Looking forward to the next decade, the expectation/aim would be to publish the same volume of surgical RCTs with a further improvement in quality.

The region of origin of published surgical RCTs has shifted, with Asia/Oceania as the leading continent in volume, whereas the number of RCTs from Europe has declined. In 2014, the new European Trials Directive started, which possibly influenced the use of RCTs in Europe^21^. In Asia, the steep increase in Chinese trials is particularly notable. China published 81 surgical RCTs in 2019, compared with 40 in 2009, and unranked a decade earlier. This was coupled with an over sixfold increase in the proportion of trials with a low risk of bias, from 2.5 per cent in 2009 to 16.0 per cent in 2019. Similar results were observed in another study that included 7422 RCTs published in Chinese medical journals and indicated that the quality of reporting surgical RCTs has improved^22–24^. This improvement could be explained by the fact that China started several programmes, such as the Thousand Talents Plan to temporarily attract scientists from abroad accompanied by government investments in innovation and healthcare^25^.

The UK had the highest proportion of trials with a low risk of bias (57.1 per cent) worldwide in 2019, in comparison to 33.3 per cent in 2009^8^. A possible explanation for this increase is the implementation of a national programme for surgical trials in the UK by the National Institute for Health Research and the Royal College of Surgeons in 2013^26^. With the advent of this programme, mentors were available to guide new research surgeons through the trial process, which could have led to the observed high methodological quality of RCTs.

Most medical and surgical journals have adopted the CONSORT criteria which has led to improved reporting of RCTs^27–32^. However, poor reporting is still common, with deficiencies in reporting the randomization method, blinding, and allocation concealment^33^. The present review showed a significant increase in the reporting of blinding, generation of allocation, and concealment of allocation in the interval 2009–2019. Nonetheless, there is still a substantial proportion of RCTs with poor quality of reporting. Journal editors and peer reviewers have a critical role in addressing this issue. This topic has received notable attention, with several published recommendations on how to improve the peer review process and appeals to encourage more journals to adopt the CONSORT criteria^34–40^.

Clearly, adequate reporting of methodological quality is not the same as actual methodological quality. Interestingly, one study^41^ even declared that the actual methodology of a published trial is often better than that reported. This study compared the study protocol with the actual published RCTs, and found that adequate allocation of concealment was achieved in all trials, but was reported in only 42 per cent of the published reports. The same was observed for the sample size calculation and the use of intention-to-treat analyses. However, users of randomized trials do not have access to the unpublished study data, making the final article essential for assessing quality.

Interestingly, this review also identified a significant shift in trial funding. Over the past 20 years, fewer RCTs have been funded by industry. This review identified an industry funding rate of 11.4 per cent in contrast with 33 per cent in another review of surgical RCTs (2008–2020)^42^. Several studies^43,44^ have shown that industry funding leads to overestimation of positive outcomes, which clearly affects the interpretation of results. On the other hand, funding of surgical RCTs may become increasingly difficult in future years with declining support from industry.

The surgical field will have to develop methods to overcome difficulties in performing surgical RCTs. Options include innovative trial designs such as registry-based trials^45–48^. Registry-based RCTs are associated with lower costs as they use an ongoing registry for data collection. Several registry-based trials are currently ongoing in the USA and Europe^48–50^. In addition, the trials within cohorts and stepped-wedge RCTs (SW-RCTs) are alternative RCT designs to overcome difficulties in surgical RCTs^51,52^. For example, a recent nationwide SW-RCT^53^ from the Netherlands, focusing on improved complication detection and management after pancreatic surgery, reported a halving of postoperative mortality.

The results of this systematic review should be interpreted in light of some limitations. First, the available Medical Subject Headings (MeSH) term titles in PubMed have changed over time^54^. This may have led to differences in the degree of identification of surgical studies between 1999, 2009, and 2019. However, the search did not rely solely on MeSH terms, but also used various permutations of free-text terms to compensate for these potential differences. Second, reports of published articles in languages not spoken fluently by the authors were excluded. This only pertained to 10 excluded RCTs from 2019 (exact languages of excluded trials are shown in *Fig. 2*). Third, trials were classified on the basis of the country of the leading department if the trial was performed across multiple countries or continents. Of the included trials, 86.8 per cent were conducted in single countries, so any possible influence of this practice on results is presumably very limited. Moreover, the majority of international trials were undertaken within the same continent. Fourth, this review is a continuation of a previously published article. The methods used were same as those employed in the earlier article, but differences in interpretation cannot be excluded. To minimize these differences, the reviewers of both studies have been in close contact to clarify any ambiguities. Fifth, although a trial with a low risk of bias is defined according to empirical evidence, it must be noted that other factors not included in this definition could influence the quality of the RCT. Therefore, *Table 3* also presents data according to other criteria to allow the reader to judge every criterion separately. Additionally, the term ‘low risk of bias’ is not the same as ‘high quality’. Rather, these trials have adequate methodology based on a number of important characteristics and, although his decreases the risk of bias, it cannot eliminate this risk completely. Sixth, there is an inevitable delay in detecting developments in surgical RCTs because of the lag time between study protocol development and final publication.

In conclusion, the volume of published surgical RCTs worldwide has remained stable in the past decade; although the reported quality improved somewhat, there remains a lot to be gained. A significant increase in volume of published surgical RCTs was observed in Asia in general and China in particular. The 10 best countries in terms of methodological quality were all from Europe, with the UK ranking first (*Table 4*). Thus, education in trial methodology, improved research infrastructure, and enforced adherence to reporting guidelines remain necessary, with additional focus on innovative trial designs to overcome the unique issues with surgical RCTs.

## Supplementary Material

znad160_Supplementary_Data

## Data Availability

Data are available on request.
