# Supplementary material for: Two decades of surgical randomized controlled trials: worldwide trends in volume and methodological quality
Source: Br J Surg. 2023 Jun 28;110(10):1300–8. doi: 10.1093/bjs/znad160 (PMC10480038; doi:10.1093/bjs/znad160)
Supplement: znad160_Supplementary_Data [file znad160_supplementary_data.zip › Supplementary_Material.docx]

**Two decades of surgical randomized controlled trials: worldwide trends in volume and methodological quality**

A.J.M. Pronk1,2, A. Roelofs1,2, D.R. Flum3, H.J. Bonjer2,4, M. Abu Hilal5, MGW Dijkgraaf6,7, M.G. Besselink1,2, U. Ahmed Ali1,2

1 Amsterdam UMC, location University of Amsterdam, Department of Surgery, Amsterdam, the Netherlands

2 Cancer Center Amsterdam, the Netherlands

3 Department of Surgery, University of Washington, Seattle, WA, USA

4 Amsterdam UMC, location Vrije Universiteit, Department of Surgery, Amsterdam, the Netherlands

5 Department of Surgery, Fondazione Poliambulanza Hospital, Brescia, Italy

6 Amsterdam UMC, location University of Amsterdam, Epidemiology and Data Science, Amsterdam, the Netherlands.

7 Amsterdam Public Health, the Netherlands

**Corresponding author:**

Prof Marc G Besselink

Amsterdam UMC, University of Amsterdam,

Department of Surgery, Cancer Center Amsterdam

De Boelelaan 1117 (ZH-7F18), 1081 HV Amsterdam, the Netherlands

[m.g.besselink@amsterdamUMC.nl](mailto:m.g.besselink@amsterdamUMC.nl)

**Supplementary Materials – Index**

**Supplementary Figures and Tables**

Table S1. Reported methodological quality per geographical region Pag. 3

Table S2. Top 10 countries by relative number of RCTs per Pag. 4

specialist surgical workforce per 100 000 inhabitants

**Supplementary Figures and Tables**

**Table S1. Reported methodological quality per geographical region**

|  | **Africa/South America** | | | | | **Asia/Oceania** | | | | | **Europe** | | | | | **North America** | | | | |
| --- | --- | --- | --- | --- | --- | --- | --- | --- | --- | --- | --- | --- | --- | --- | --- | --- | --- | --- | --- | --- |
| **Methodological Quality Characteristic** | **1999 (n=6)** | **2009 (n=31)** | **RR (95 per cent CI)^#^** | **2019 (n=30)** | **RR (95 per cent CI)^#^** | **1999 (n=61)** | **2009 (n=159)** | **RR (95 per cent CI)^#^** | **2019 (n=199)** | **RR (95 per cent CI)^#^** | **1999 (n=161)** | **2009 (n=204)** | **RR (95 per cent CI)^#^** | **2019 (n=154)** | **RR (95 per cent CI)^#^** | **1999 (n=72)** | **2009 (n=56)** | **RR (95 per cent CI)^#^** | **2019 (n=55)** | **RR (95 per cent CI)^#^** |
| **1. Primary outcome explicitly stated** | 33 | 42 | 1.26 (0.38–4.20) | 73 | 0.57 (0.36-0.91) | 62.3 | 50.9 | 0.82 (0.64–1.05) | 59.8 | 0.85 (0.70-1.03) | 72.0 | 77.9 | 1.09 (0.97–1.23) | 83.1 | 0.94 (0.85-1.04) | 65.3 | 76.8 | 1.18 (0.94–1.47) | 83.6 | 0.92 (0.76-1.11) |
| **2. Sample size calculation described** | 17 | 23 | 1.36 (0.20–9.10) | 70 | 0.32 (0.16-0.65) | 27.9 | 33.9 | 1.22 (0.77–1.93) | 56.8 | 1.55 (1.27-1.88) | 36.0 | 58.8 | 1.65 (1.30–2.08) | 76.0 | 0.78 (0.68-0.90) | 33.3 | 64.3 | 1.85 (1.28–2.69) | 80.0 | 0.80 (0.64-1.02) |
| **3. Baseline present** | 50 | 87 | 1.74 (0.77–3.92) | 97 | 0.90 (0.78-1.05) | 95.1 | 91.2 | 0.96 (0.89–1.03) | 93.0 | 0.99 (0.93-1.05) | 87.6 | 94.1 | 1.07 (0.99–1.14) | 94.1 | 1.00 (0.95-1.05) | 95.8 | 89.3 | 0.93 (0.84–1.03) | 98.2 | 0.91 (0.83-1.00) |
| **4. Generation of allocation: reported and adequate** | 17 | 42 | 2.52 (0.40–15.8) | 70 | 0.60 (0.37-0.96) | 24.6 | 35.2 | 1.41 (0.86–2.29) | 62.8 | 0.55 (0.43-0.70) | 31.7 | 55.9 | 1.73 (1.34–2.23) | 66.9 | 0.84 (0.71-0.99) | 38.9 | 55.4 | 1.42 (0.98–2.07) | 61.8 | 0.90 (0.65-1.23) |
| **5. Concealment of allocation: reported and adequate** | 17 | 52 | 3.10 (0.50–19.1) | 77 | 0.67 (0.45-1.00) | 34.4 | 37.7 | 1.11 (0.75–1.66) | 56.8 | 0.68 (0.54-0.85 | 34.1 | 59.8 | 1.78 (1.40–2.79) | 72.1 | 0.83 (0.72-0.96) | 27.8 | 44.6 | 1.61 (1.00–2.58) | 72.7 | 0.61 (0.44-0.86) |
| **6. Blinding: any type of blinding** | 0,0 | 23 | NA | 47 | 0.48 (0.23-1.03) | 27.9 | 27.0 | 0.97 (0.60–1.57) | 35.2 | 0.78 (0.57-1.07) | 29.1 | 28.9 | 0.76 (0.57–1.02) | 37.7 | 0.77 (0.57-1.03) | 33.3 | 50.0 | 1.50 (0.99–2.28) | 70.9 | 0.71 (0.52-0.96) |
| **7. Double-blinding stated** | 0,0 | 10 | NA | 20 | 2.07 (0.57-7.52) | 14.8 | 18.2 | 1.24 (0.62–2.46) | 12.1 | 0.66 (0.40-1.09) | 26.7 | 19.1 | 0.68 (0.47–1.00) | 16.9 | 0.91 (0.58-1.43) | 26.3 | 35.7 | 1.35 (0.80–2.81) | 27.3 | 0.76 (0.44-1.33) |
| **8. Intention to treat analyses** | 17 | 13 | 0.77 (0.10–5.77) | 10 | 0.78 (0.19-3.18) | 14.85 | 18.2 | 1.19 (0.60–2.38) | 27.1 | 1.51 (1.01-2.27) | 24.2 | 48.0 | 2.02 (1.47–2.76) | 51.9 | 1.09 (0.89-1.35) | 16.7 | 35.7 | 2.14 (1.15–4.00) | 36.4 | 1.02 (0.62-1.67) |
| **9. Handling of dropouts adequate** | 67 | 77 | 1.21 (0.67–2.19) | 60 | 0.78 (0.55-1.10) | 78.7 | 81.1 | 1.02 (0.89–1.17) | 6059.8 | 0.75 (0.65-0.86) | 83.2 | 84.8 | 1.01 (0.93–1.09) | 68.1 | 0.79 (0.70-0.90) | 80.5 | 83.9 | 1.03 (0.90–1.19) | 74.5 | 0.87 (0.71-1.06) |
| **10. Low risk of bias*** | 0.0 | 9.7 | NA | 6.7 | 0.69 (0.12-3.4) | 4.9 | 5.0 | 1.02 (0.28–3.73) | 18.1 | 3.50 (1.70-7.32) | 7.5 | 23.0 | 3.03 (1.65–5.52) | 30.5 | 1.35 (0.96-1.92) | 2.8 | 16.1 | 5.79 (1.30–25.7) | 23.6 | 1.47 (0.69-3.16) |
|  | Values in parentheses are percentages unless indicated otherwise  *Trial with adequate generation of allocation, adequate concealment of allocation, intention-to-treat analyses, and adequate handling of dropouts.  ^#^This comparison was performed in our previous study.^8^  CI indicates confidence interval; NA, not applicable; RR, relative rate. | | | | | | | | | | | | | | | | | | | |

**Table S2. Top 10 countries by relative number of RCTs per specialist surgical workforce per 100 000 inhabitants**

| **Rank** | **Country** | **No. Trials** | **No. Of Trials per specialist surgical workforce per 100 000 inhabitants*** |
| --- | --- | --- | --- |
| **1** | Nigeria | 2 | 4,9 |
| **2** | China | 81 | 2,0 |
| **3** | India | 11 | 1,6 |
| **4** | Iran | 8 | 1,6 |
| **5** | Pakistan | 7 | 1,3 |
| **6** | Japan | 36 | 1,0 |
| **7** | USA | 50 | 0,9 |
| **8** | Netherlands | 27 | 0,6 |
| **9** | Sri Lanka | 1 | 0,4 |
| **10** | Egypt | 18 | 0,4 |
|  | *No. Of trials per specialist surgical workforce per 100 000 inhabitants based on the available data (applying to 2014-2018) on <https://ourworldindata.org/grapher/surgeons-per-100000?tab=table> | | |
